# Sex-Dependent Impact of Low-Level Lead Exposure during Prenatal Period on Child Psychomotor Functions

**DOI:** 10.3390/ijerph15102263

**Published:** 2018-10-16

**Authors:** Kinga Polanska, Wojciech Hanke, Natalia Pawlas, Ewelina Wesolowska, Agnieszka Jankowska, Marta Jagodic, Darja Mazej, Jolanta Dominowska, Mariusz Grzesiak, Fiorino Mirabella, Flavia Chiarotti, Gemma Calamandrei

**Affiliations:** 1 Department of Environmental Epidemiology, Nofer Institute of Occupational Medicine, 91-348 Lodz, Poland; wojciech.hanke@imp.lodz.pl (W.H.); ewelina.wesolowska@imp.lodz.pl (E.W.); agnieszka.jankowska@imp.lodz.pl (A.J.); 2 Chair and Department of Pharmacology, Medical University of Silesia, School of Medicine with Division of Dentistry in Zabrze, 41 808 Zabrze, Poland; n-pawlas@wp.pl; 3 Department of Environmental Sciences, Jožef Stefan Institute, SI-1000 Ljubljana, Slovenia; marta.jagodic@ijs.si (M.J.) darja.mazej@ijs.si (D.M.); 4 Department of Teaching Midwifery, Medical University of Lodz, 90-419 Lodz, Poland; jolad48@wp.pl; 5 Obstetrics, Perinatology and Gynecology Department, Polish Mother’s Memorial Hospital Research Institute, 93-338 Lodz, Poland; mariusz.grzesiak@gmail.com; 6 Center for Behavioral Sciences and Mental Health, National Institute of Health, I-00161 Rome, Italy; fiorino.mirabella@iss.it (F.M.); flavia.chiarotti@iss.it (F.C.); gemma.calamandrei@iss.it (G.C.)

**Keywords:** sex differences, cord blood lead level, prenatal exposure, neurodevelopment, cognitive, language and motor functions

## Abstract

The impact of exposure to lead on child neurodevelopment has been well established. However, sex differences in vulnerability are still not fully explained. We aimed at evaluating the effect of a low-level lead exposure, as measured between 20 to 24 weeks of pregnancy and in cord blood, on developmental scores up to 24 months of age in 402 children from the Polish Mother and Child Cohort (REPRO_PL). Additionally, sex-dependent susceptibility to lead at this very early stage of psychomotor development was assessed. The blood lead levels were analyzed using inductively coupled plasma mass spectrometry (ICP-MS). In order to estimate the children’s neurodevelopment, the Bayley Scales of Infant and Toddler Development was applied. The geometric mean (GM) for blood lead level during 20–24 weeks of pregnancy was 0.99 ± 0.15 µg/dL and, in the cord blood, it was 0.96 ± 0.16 µg/dL. There was no statistically significant impact of lead exposure during prenatal period on the girls’ psychomotor abilities. Among the boys, we observed lower scores for cognitive functions, along with increasing cord blood lead levels (β = −2.07; *p* = 0.04), whereas the results for the language and motor abilities were not statistically significant (*p* > 0.05). Our findings show that fetal exposure to very low lead levels might affect early cognitive domain, with boys being more susceptible than girls. Education on health, higher public awareness, as well as intervention programs, along with relevant regulations, are still needed to reduce risks for the vulnerable population subgroups.

## 1. Introduction

Harmful effects of lead, especially in children, are one of the biggest public health concerns worldwide [1,2,3,4,5,6]. It is not under discussion that this metal can cross the blood–placenta and blood–brain barriers, resulting in neurodevelopmental toxicity during the prenatal period [7,8,9]. It also needs to be emphasized that lead exposure has decreased significantly over the last decades. This is a consequence of removing lead from gasoline and paints, and implementing occupational and environmental safety measures [1,2,3,4,5,6]. As an example, the lead level in blood of pre-school children in the United States dropped from 15 µg/dL, reported in the 1970s, to 1.9 µg/dL, at the beginning of this century. However, there are still approximately half a million U.S. children aged 1–5 with blood lead levels above 5 µg/dL [1,10]. In Poland, in Legnica-Glogow Copper Mining District, the blood lead level in children dropped from 10 µg/dL in 1991, to 4 µg/dL in 2009 [11,12]. According to data from the REPRO_PL cohort, the mean lead level equaled 1.1 µg/dL in blood collected between 20 to 24 weeks of pregnancy, and 1.0 µg/dL in the cord blood with none of the women having levels above 6.0 µg/dL [12,13]. Despite the decreasing level of this metal in the environment, exposure to low lead levels (blood lead levels below 5 µg/dL) continues to be widespread in the general population.

Although the blood lead level of concern for neurodevelopmental toxicity in children decreased from 60 µg/dL in the 1960s to 10 µg/dL in 1991, and 5 µg/dL in 2012, epidemiological and experimental data indicate neurological as well as neuropsychological effects, also, well below this level [1,2,3,4,5,6,10]. Furthermore, the induced neurodevelopmental impairment might persist into adulthood [14,15].

Much evidence demonstrates differences between sexes with regard to the patterns of exposure, chemical absorption, characteristics of metabolism, and ability to detoxify [16,17]. Sexual hormones influence sexually dimorphic development of the brain. Estrogen receptors are distributed and located differently in males and females, which is associated with gender-specific neurotoxicity. Estrogens play a crucial role in the regulation of neuronal structure and protection of brain against oxidative damage. Also, the disrupting capability of thyroid homeostasis differs by gender, and thyroid hormone is the one which is critical in terms of brain development [18].

Animal studies indicate that prenatal lead exposure produces more marked deficits in the male sex [19]. In addition, most of the studies on humans have reported stronger harmful effects of lead exposure in males than in females [18,20,21,22,23,24]. Nevertheless, there are some that indicate no difference between the genders [25,26,27].

Still, one has to remember that the majority of the studies in this field have considered sex as one of the confounders, rather than as an effect modifier, and this hampered any definitive conclusion on possible sex differences in vulnerability to the neurodevelopmental impact of lead exposure [17].

Our goal was twofold: (i) to evaluate the effect of a low-level lead exposure as measured between 20 to 24 weeks of pregnancy, and in cord blood on developmental scores up to 24 months of age, in the children from the Polish Mother and Child Cohort (REPRO_PL), and (ii) to assess sex-dependent susceptibility to lead at this very early stage of psychomotor development.

## 2. Material and Methods

### 2.1. Design of the Study and Analyzed Population

Description of the REPRO_PL cohort, including study design, population, and analyzed variables, has been published in other papers [28,29]. This prospective cohort was created in 2007 to evaluate pre- and postnatal exposure to the factors related to the environment and lifestyle, and their impact on outcomes of pregnancy, as well as health status and neurodevelopment of the children.

The women met with a midwife three times in pregnancy and after the delivery, in order to collect questionnaire data, as well as biological samples. Twelve and 24 months after the delivery, the mothers and their children participated in the second phase of the study covering child exposure, health status, and neurodevelopment assessment [29].

Altogether, 539 children underwent an examination at least once within two years after their birth. Two hundred and eighty samples of the blood, collected during the 2nd trimester of pregnancy, and 303 cord blood samples, were randomly selected for the analysis (this constitutes 402 subjects with at least one measurement (74.6% of the original sample)).

The study obtained approval from the Ethical Committee of the Nofer Institute of Occupational Medicine (NIOM), Lodz, Poland (No. 7/2007 and No. 3/2008). All of the study subjects provided their written, informed consents.

### 2.2. Blood Lead Level Measurements

The blood samples were collected by venipuncture using S-Monovette with lithium heparin as an anticoagulant, and frozen at −20 °C until the analysis. Blood lead levels were analyzed in two laboratories: the Laboratory of Metal Analysis of the Nofer Institute of Occupational Medicine (NIOM) in Lodz, Poland (*N* = 535) and the laboratory of Jožef Stefan Institute (JSI), Ljubljana, Slovenia (*N* = 48). No significant differences between NIOM and JSI analyses were noted (*p* > 0.05).

The method used by NIOM (with the limit of detection of 0.16 ng/mL) has been described, in detail, in our previous publications [12,13]. In JSI, the blood samples (300 μL) were first digested by the addition of 500 μL of 65% nitric acid (Suprapur) using the microwave system (ULTRAWAVE, MILESTONE Srl, Sorisole, Italy), followed by measurement of lead using the Triple Quadrupole Inductively Coupled Plasma Mass Spectrometer (ICP-QQQ, Agilent 8800, Tokyo, Japan). The limit of detection was 1.7 ng/g. Quality of the results was checked via the regular use of the reference material Seronorm^TM^ Whole Blood Level 1 (SERO, Billingstad, Norway) and participation in German External Quality Assessment Scheme (G-EQUAS, Erlangen-Nuremberg, Germany).

### 2.3. Neurodevelopment Assessment

In order to estimate the children’s neurodevelopment (including language, cognitive, and motor functions) at the age of one and two, the Bayley Scales of Infant and Toddler Development (Bayley 3rd edition) was applied [13,30,31,32].

### 2.4. Covariates

A detailed description of the covariates has been published previously [32]. The following variables were considered: parental age and educational level, socioeconomic (SES) and marital status, sex of the child, complications during pregnancy, delivery type, week of pregnancy, and anthropometric measures at birth, breastfeeding, number of kids at home, day care attendance, cotinine level in saliva, and alcohol consumption during pregnancy, as well as child environmental tobacco smoke (ETS) exposure after birth. All variables with *p* < 0.10 were included into the model.

### 2.5. Statistical Analysis

At first, for the purpose of the analyses, we calculated frequencies for qualitative variables and means ± SD for quantitative variables. Secondly, a correlation between 2nd trimester and cord blood lead levels (both log10 transformed) was computed by the Pearson correlation coefficient, in the overall group of children, and in males and females, separately. Then, we conducted separate analyses for the multivariate linear regression using cognitive, language, and motor developmental scores as dependent variables, and 2nd trimester and cord blood lead levels as independent variables. The regression models included covariates such as person who conducted child examination, age and educational level of the mothers, child sex and age, and cotinine level in saliva collected during pregnancy. Finally, based on the result of the previous analysis, separate analyses for the males and females were performed. The 2nd trimester and cord blood lead levels, as well as the cotinine levels, were log10 transformed, and all the quantitative variables (including log10-transformed blood lead levels and cotinine) were standardized before the regression analysis. We calculated the variance inflation factor (VIF) for each variable in each model, so as to verify multicollinearity presence among the explanatory variables. The corresponding independent variables were excluded from the model in the case of VIF values higher than 4. The Huber/White/sandwich estimator of variance was applied to estimate standard errors of regression coefficients, in order to account for dependence between observations coming from the subjects undergoing the Bayley assessment at both 12 and 24 months of age. We reported regression coefficients, along with their 95% confidence interval and a statistical significance level (*p*-value). Based on the number of measurements of blood lead levels, at the 2nd trimester (300, 142 and 158, for all, males, and females, respectively) and/or on cord blood (365, 190, and 175, for all, males, and females, respectively), our study could detect small size effects of lead level (Cohen f2 ≤ 0.056 in the total group of children, and in males and females, separately) on cognitive, language, and motor domains, in a multiple regression analysis with a power equal 0.80 and a significance level equal 0.05. Finally, in order to verify if the effect of blood lead levels on Bayley scores could be affected by the blood lead levels value, scatterplots of blood lead levels vs Bayley scores were visually inspected, and the regression analyses of blood lead levels on Bayley scores were repeated in the subgroups of children based on the quartiles of blood lead levels. SPSS Statistics 25 (Statistical Package for Social Science) and STATA 8.1 software (StataCorp, College Station, TX, USA) were applied for the purpose of the statistical analyses.

## 3. Results

### 3.1. Characteristic Features of the Mothers and Children

Characteristic features of the mothers and children were described, in detail, in our previous publications, and are presented in the Supplementary Materials (Table S1) [13,30,31,32]. In short, a significant part of the mothers had university education (70.1%), were married (77.1%), and belonged to medium or high SES groups (91.1%).

Mean composite scores for language, cognitive, and motor functions represented an average or a high-average level (Table 1). The lead level in the blood collected during the 2nd trimester of pregnancy ranged from 0.29 to 2.63 µg/dL, with a geometric mean (GM) of 0.99 µg/dL (geometric standard deviation (GSD) ± 0.15 µg/dL). For the cord blood, the range of lead level was 0.24–5.65 µg/dL, with only one sample above 5 µg/dL (GM = 0.96 µg/dL; GSD ± 0.16 µg/dL). There were no significant differences in the mean lead levels in the blood between the girls and boys (*p* > 0.5).

### 3.2. Correlation between 2nd Trimester and Cord Blood Lead Levels and Association between Lead Level in the Blood and Child Neurodevelopment

The correlation between the 2nd trimester and cord blood lead levels was low to moderate: specifically, Pearson r was equal 0.37 (*N* = 181, *p* < 0.001), 0.44 (*N* = 90, *p* < 0.001), and 0.29 (*N* = 91, *p* < 0.005) in the overall group of children, and in males and females, respectively. Results of the multivariate linear regression analysis for the impact of selected sociodemographic, lifestyle variables, and cord blood lead level on child development, are presented in the Supplementary Materials (Tables S2 and S3). The regression analyses, evaluating the effect of cord blood lead level on cognitive, language, and motor scores by excluding the subjects having a lead level greater than or equal 4.0 µg/dL and 3.0 µg/dL, are presented in Tables S4 and S5. There were no substantial differences in the results with respect to the analysis performed on all the subjects. Generally, there was no interaction for blood lead levels and sex; however, for cord blood lead levels and sex, it was quite close to significance (*p* = 0.1). The strong effect of sex in psychomotor domains justifies the separate analyses for the males and females. The results of the multivariate analysis presenting sex differences in the effects of lead exposure during prenatal period on child psychomotor abilities, are included in Table 2. There was no statistically significant impact of prenatal lead exposure on the girls’ psychomotor development. However, for the boys, we observed lower scores for cognitive functions, along with increasing cord blood lead levels (β = −2.07; *p* = 0.04). For the language and motor abilities, the results were not statistically significant (*p* > 0.05).

Visual inspection of scatterplots of blood lead levels (log10-transformed) vs Bayley scores did not evidence any clear non-linear relationship between the variables. This was also supported by the regression analyses performed separately in the subgroups of children based on quartiles of blood lead levels. Indeed, the regression coefficients computed in the four subgroups were not significantly different from zero for most of the combinations of blood lead level values by Bayley domain by age at assessment, and their confidence intervals greatly overlapped across quartiles for most combinations (Figures S1 and S2).

## 4. Discussion

This prospective mother–child cohort study indicates that low-level lead exposure during the prenatal period is negatively associated with cognitive scores within the first two years of life in the male sex only. Although huge improvements have been made regarding exposure to lead in the general population, our findings, together with existing research, underline that education on health, higher public awareness, as well as intervention programs, along with relevant regulations, are still needed.

As mentioned above, maternal blood lead levels correlate with fetal ones [33]. It is well known that the increase in calcium requirements during pregnancy enhances Ca^++^ bone turnover, with a subsequent release into the bloodstream of lead stored in bones [18,24]. As evidenced in animal models, lead easily crosses the blood–brain barrier and reaches the developing brain, interfering with synaptic maturation and functioning of different mechanisms [34]. In our study, the geometric mean for blood lead level during 20–24 weeks of pregnancy was 0.99 ± 0.15 µg/dL, and in the cord blood it was 0.96 ± 0.16 µg/dL. Blood lead levels in the cord blood, consistent with our study, were reported in Poland by Jedrychowski et al. (2009) (median 1.21 µg/dL; range 0.44–4.60 µg/dL) [21] and in Korea by Joo et al. (2018) (geometric mean 0.90 ± 1.57 µg/dL) [18]. In the Avon Longitudinal Study of Parents and Children (ALSPAC), the mean lead level in the blood collected in the 1st trimester of pregnancy was 3.67 ± 1.46 (range 0.20–19.14) µg/dL, which was higher than that observed in our study population [24]. In the earlier study by Dietrich et al. (1987), the arithmetic mean of lead concentration in umbilical cord blood was 6.3 ± 4.5 µg/dL [27]. The analyses performed previously on REPRO_PL cohort indicated the following predictors of environmental lead exposure among pregnant women in Poland: less years of education, older age, higher BMI before pregnancy, smoking status, as well as smaller distance from the place of residence to the copper smelter (in a subpopulation of the study sample who lived in the region where copper smelters were located) [12]. These factors need to be considered when creating educational and interventional activities.

Our present results add to the large body of evidence showing that even in the case of very low levels of exposure to lead during pregnancy, the maturation of cognitive capacities in the first years of life is specifically impaired.

Most of the existing epidemiological studies have focused on the impact of postnatal lead exposure on child neurodevelopment, and the impact of prenatal exposure on the outcome of interest has been investigated to a lower extent [35]. Such studies differ in the design of the study and participant’s characteristics (including the size of the study groups); timing of blood collection (early, late pregnancy, cord blood) and exposure level; timing and analyzed domains of child neurodevelopment; statistical methods applied; and confounding factors included in the analysis [18,24]. However, altogether, the studies indicate a negative impact of lead exposure during the prenatal period on child development, regardless of the level of exposure, and emphasize the absence of a lower safety boundary [1,24].

Similarly to our results, a few other prospective studies, also including a prospective birth cohort established in Poland, have found that the detrimental effects of prenatal low-level lead exposure are more marked in boys than in girls [18,21,23,24,27].

Sex-dependent neurotoxic effects of metals, and of lead in particular, have been identified in a few cohorts [17]. Dietrich et al. (1987) showed that boys of 3 and 6 months of age obtained worse results in the Bayley mental developmental index, and there was a negative association between those results and prenatal and neonatal blood lead levels. However, in that population, the mean lead level in the cord blood was much higher (6.3 ± 4.5 µg/dL) than in the current study. Additional analyses have demonstrated that those effects partly depended on a lower birth weight and pregnancy disturbances related to lead [27]. A further observation in that cohort confirmed a significant interaction between gender and lead exposure biomarkers in the participants when they were assessed at adolescence (15–17 years old), with worse results of neuropsychological tests in males [23]. In an analysis of a prospective cohort of 444 3-year-old children, the authors have shown that even a very low exposure to lead during prenatal period results in a significant inverse association with mental development, with lower cognitive abilities in boys than in girls [22]. In 457 36-month-old children, the same researchers have found that prenatal lead exposure was inversely associated with the Bayley mental development index in boys while, in girls, there was no significant effect [21]. In the study by Taylor et al. (2017) in ALSPAC cohort, there was no association in the adjusted models between prenatal blood lead levels and IQ results for 4- and 8-year-old children, in this same cohort the interaction between gender and blood lead was not significant in 4-year olds, while it appeared to be of borderline significance in 8-year-old children for verbal performance and total IQ score, and boys were more susceptible [24]. Finally, the study by Joo et al. (2018) in a prospective cohort has confirmed that late pregnancy lead levels are significantly related to behavioral disturbances assessed by the Child Behavior Checklist in boys, while girls are more vulnerable to lead exposure after birth [18].

A large body of data shows that environmental cues, integrated with gonadal sex and varying hormone levels during development, alter the network of molecular interactions, which eventually results in sex-specific phenotypes, including sex dimorphism in cognitive functions in typical children. Within such a dynamic framework, the reasons for sex-dependent vulnerability to developmental lead exposure are far from clear. Recent studies have confirmed that effects of a low dose lead exposure on associative end points are modulated by both sex and timing of exposure [36]. Animal experiments point to several potential mechanisms implicated, including sex dimorphism in gene expression patterns in the developing brain [37], and sex-related differences in antioxidant defenses [38,39] and neuroendocrine immune networks [40]. The protective role of estrogens on neural growth and oxidative stress responses might, in part, explain the greater vulnerability of male sex to heavy metals such as methylmercury and lead [41,42].

Increasing evidence points to sex differences in epigenetic mechanisms: DNA methylation profiles in brain can be modified by hormonal influences [42,43,44,45,46]. A recent study by Singh et al. (2018) found that rats exposed to different lead doses, during different developmental windows, displayed significant alterations of methylation profiles in genes functionally relevant to brain development, notably, the lead-induced changes of DNA methylation in the hippocampus were markedly sex-dependent [47]. These findings are consistent with the result of a research performed in peripheral blood samples from children of both sexes, where lead-associated changes in DNA methylation showed a significant sex dimorphism [48]. The increasing number of studies showing that developmental exposure to metals influences DNA methylation in exposed children supports the view that the long-term effects of these agents might be mediated by epigenetic changes [49]. Recently, epigenetic effects of prenatal lead exposure have been also demonstrated in children; however, there have been no marked differences between the sexes—gender was treated as a confounding factor [50].

Strengths and weaknesses of the present study need to be mentioned. One of the advantages of the current study is the study design (a population-based prospective birth cohort) and inclusion of a variety of confounders, as well as the use of the Bayley test for neuropsychological assessment in young children. Additionally, in the current analysis, we also considered timing of the exposure (2nd trimester of pregnancy and cord blood) that may be critical in relation to developmental time points and consequent adverse effects. Notwithstanding the similar exposure levels, effects are evident only for cord blood lead exposure [18,24].

The major drawback of the study is the fact that there was no measurement of lead levels in the blood collected from the children which, considering their young age (up to 2 years of life) was not possible in the REPRO_PL cohort. Then, despite the fact that the analysis covered a variety of covariates, we did not consider the quality of home environment, relationship between the mother and her child, parental roles, or maternal IQ. In the case of most of these variables, SES and the level of maternal education can be regarded a reliable proxy (confounding effect of maternal education, SES, number of brothers and sisters, and day care attendance as surrogate were considered) [31]. In addition, the possibility of selection bias has to be discussed. In that respect, it needs to be pointed out that the study sample is representative of an urban population in Poland (with a significant proportion of women indicating a high SES, university degree, and marital status). However, it cannot be representative of rural or disadvantaged areas. As was mentioned above, the maternal educational level (also as the proxy of SES) has been included in the regression model. Additionally, the analysis looking at the lead level and scores evaluating cognitive, language, and motor functions in a low-medium vs high SES was performed. There were no statistically significant differences between a low-medium and high SES with respect to lead levels, either in the 2nd trimester of pregnancy or in the cord blood, as well as cognitive, language, and motor abilities at 1 or 2 years of age (data not shown). Finally, another minus of the study is associated with the lack of measurement/or inclusion of exposure to neurotoxic elements, such as As, Cd, or Hg, which can act in synergy with lead or even counteract its effects on neural development. The Shah-Kulkarni et al. study (2016) indicates that iron status in pregnancy may play a significant part in modifying lead exposure effects on the fetus [20]. More studies on potential confounding and/or modifying effects due to a mixed exposure to various neurotoxicants, as well as protective factors, should be performed in the future [18,24].

## 5. Conclusions

Our findings show that fetal exposure to very low lead levels might affect the early cognitive domain, with boys being far more susceptible than girls. There are plenty of epidemiological studies on human health risk assessment dealing with toxicological neurodevelopmental effects, but most of them include sex as a confounding factor, rather than as an effect modifier. Vahter et al. (2007) advised planning and designing environmental studies to include sex and gender-based analyses [51]. Higher public awareness of the prenatal life stage importance, education on health, as well as intervention programs, along with relevant regulations, still have to be considerably enhanced, in order to reduce risks for the vulnerable population subgroups.

## Figures and Tables

**Table 1:** Characteristics of the exposure and outcomes variables.

Variables	GM	GSD	Mean	SD	Median	Min	Max
Blood lead level in the 2nd trimester of pregnancy (μg/dL)							
All subjects; *N* = 280	0.99	0.15	1.09	0.46	1.01	0.29	2.63
Males; *N* = 130 *	1.01	0.15	1.10	0.45	1.07	0.34	2.59
Females; *N* = 150	0.97	0.16	1.07	0.47	0.98	0.29	2.63
Cord blood lead level (μg/dL)							
All subjects; *N* = 303	0.96	0.16	1.09	0.65	0.93	0.24	5.65
Males; *N* = 155 **	0.99	0.16	1.11	0.59	0.94	0.29	4.04
Females; *N* = 148	0.94	0.16	1.08	0.71	0.92	0.24	5.65
Composite score for the one-year-old children; *N* = 367							
Cognitive	-	-	106.9	10.3	105	80	145
Language	-	-	108.6	13.6	109	68	141
Motor	-	-	105.4	13.9	107	73	151
Composite score for the two-year-old children; *N* = 233							
Cognitive	-	-	112.6	16.3	110	80	145
Language	-	-	102.1	12.8	100	74	144
Motor	-	-	112.0	14.4	110	73	154

Comparison between males and females * *t*-test *p* = 0.56; ** *t*-test *p* = 0.68. GM—geometric mean. GSD—geometric standard deviation. SD—standard deviation.

**Table 2:** The 2nd trimester and cord blood lead level and child psychomotor abilities.

Lead Level (**µ**g/dL)	Child Sex	Cognitive			Language			Motor		
		β	95% CI	*p*	β	95% CI	*p*	β	95% CI	*p*
2nd trimester of pregnancy	Females ^a^	0.31	−2.00; 2.61	0.79	−1.24	−3.28; 0.79	0.23	−1.16	−3.26; 0.94	0.27
	Males ^b^	0.73	−1.19; 2.65	0.45	−0.48	−2.53; 1.57	0.64	0.48	−1.55; 2.52	0.64
Cord blood	Females ^c^	0.34	−1.30; 1.98	0.68	−0.29	−2.23;1.65	0.77	0.48	−1.55; 2.52	0.64
	Males ^d^	−2.07	−4.07; −0.06	0.04	−0.43	−2.81;1.95	0.72	−0.70	−2.90; 1.51	0.53

^a^*N* subjects = 106, *N* observations = 158; ^b^ *N* subjects = 94, *N* observation = 142; ^c^ *N* subjects = 114, *N* observations = 175; ^d^ *N* subjects = 124, *N* observation = 190. Coefficients, 95% CI—95% confidence interval, *p*-values; the 2nd trimester, cord blood lead levels, and cotinine levels during pregnancy were log10 transformed and standardized.

## Supplementary Materials

The following are available online at http://www.mdpi.com/1660-4601/15/10/2263/s1, Table S1. The child and parental characteristics; Table S2. Impact of selected sociodemographic, lifestyle variables and 2nd trimester blood lead level on the child psychomotor development within the first two years of life (*N* subjects = 200, *N* observations = 300); Table S3. Impact of selected sociodemographic, lifestyle variables, and cord blood lead level on the child psychomotor development within the first two years of life (all subjects; *N* subjects = 238, *N* observations = 365); Table S4. Impact of selected sociodemographic, lifestyle variables, and cord blood lead level on the child psychomotor development within the first two years of life (excluding subjects with cord blood lead level ≥4.0 µg/dL; *N* subjects = 235, *N* observations = 359); Table S5. Impact of selected sociodemographic, lifestyle variables, and cord blood lead level on the child psychomotor development within the first two years of life (excluding subjects with cord blood lead level ≥3.0 µg/dL; *N* subjects = 233, *N* observations = 356); Figure S1. Scatterplots relating blood lead levels in the 2nd trimester of pregnancy and in the cord blood to the Bayley cognitive, language, and motor scores at one and two years of age; Figure S2. Point estimates and 95% confidence intervals of regression coefficients measuring the effect of blood lead level (log10-transformed and standardized) on Bayley scores in the subgroups of children based on quartiles of blood lead level.

## References

[1] Bellinger D.C. (2008). Very low lead exposures and children’s neurodevelopment. Curr. Opin. Pediatr..

[2] Bellinger D.C. (2004). Lead. Pediatrics.

[3] Canfield R.L., Henderson C.R., Cory-Slechta D.A., Cox C., Jusko T.A., Lanphear B.P. (2003). Intellectual impairment in children with blood lead concentrations below 10 microg per deciliter. N. Engl. J. Med..

[4] Lanphear B.P., Hornung R., Khoury J., Yolton K., Baghurst P., Bellinger D.C., Canfield R.L., Dietrich K.N., Bornschein R., Greene T. (2005). Low-level environmental lead exposure and children’s intellectual function: An international pooled analysis. Environ. Health Perspect..

[5] Crump K.S., Van Landingham C., Bowers T.S., Cahoy D., Chandalia J.K. (2013). A statistical reevaluation of the data used in the Lanphear et al. (2005) pooled-analysis that related low levels of blood lead to intellectual deficits in children. Crit. Rev. Toxicol..

[6] Grandjean P. (2010). Even low-dose lead exposure is hazardous. Lancet.

[7] Gershanik J.J., Brooks G.G., Little J.A. (1974). Blood lead values in pregnant women and their offspring. Obstet. Gynecol..

[8] Rudge C.V., Rollin H.B., Nogueira C.M., Thomassen Y., Rudge M.C., Odland J.O. (2009). The placenta as a barrier for toxic and essential elements in paired maternal and cord blood samples of South African delivering women. J. Environ. Monit..

[9] Basha C.D., Reddy R.G. (2015). Long-term changes in brain cholinergic system and behavior in rats following gestational exposure to lead: Protective effect of calcium supplement. Interdiscip. Toxicol..

[10] Centers for Disease Control and Prevention (2018). What Do Parents Need to Know to Protect Their Children?. http://www.cdc.gov/nceh/lead/ACCLPP/blood_lead_levels.htm.

[11] Strugała-Stawik H., Rudkowski Z., Pastuszek B., Morawiec K. (2010). Biomonitoring of lead in blood of children—Short assessment of results 1991–2009. Environ. Med..

[12] Polańska K., Hanke W., Sobala W., Trzcinka-Ochocka M., Ligocka D., Strugała-Stawik H., Magnus P. (2014). Predictors of environmental lead exposure among pregnant women—A prospective cohort study in Poland. Ann. Agric. Environ. Med..

[13] Polanska K., Hanke W., Sobala W., Trzcinka-Ochocka M., Ligocka D., Brzeznicki S., Strugala-Stawik H., Magnus P. (2013). Developmental effects of exposures to environmental factors: The Polish Mother and Child Cohort Study. Biomed. Res. Int..

[14] Obeng-Gyasi E. (2018). Lead Exposure and Oxidative Stress-A Life Course Approach in US Adults. Toxics.

[15] Reuben A., Caspi A., Belsky D.W., Broadbent J., Harrington H., Sugden K., Houts R.M., Ramrakha S., Poulton R., Moffitt T.E. (2017). Association of childhood blood lead levels with cognitive function and socioeconomic status at age 38 years and with IQ change and socioeconomic mobility between childhood and adulthood. JAMA.

[16] Gochfeld M. (2007). Framework for gender differences in human and animal toxicology. Environ. Res..

[17] Llop S., Lopez-Espinosa M., Rebagliato M., Ballester F. (2013). Gender differences in the neurotoxicity of metals in children. Toxicology.

[18] Joo H., Choi J.H., Burm E., Park H., Hong Y.C., Kim Y., Ha E.H., Kim Y., Kim B.N., Ha M. (2018). Gender difference in the effects of lead exposure at different time windows on neurobehavioral development in 5-year-old children. Sci. Total. Environ..

[19] Leasure J.L., Giddabasappa A., Chaney S., Johnson J.E., Pothakos K., Lau Y.S., Fox D.A. (2008). Low-level human equivalent gestational lead exposure produces sex-specific motor and coordination abnormalities and late-onset obesity in year-old mice. Environ. Health Perspect..

[20] Shah-Kulkarni S., Ha M., Kim B.M., Kim E., Hong Y.C., Park H., Kim Y., Kim B.N., Chang N., Oh S.Y. (2016). Neurodevelopment in early childhood affected by prenatal lead exposure and iron intake. Medicine.

[21] Jedrychowski W., Perera F.P., Jankowski J., Mrozek-Budzyn D., Mroz E., Flak E., Edwards S., Skarupa A., Lisowska-Miszczyk I. (2009). Gender specific differences in neurodevelopmental effects of prenatal exposure to very low-lead levels: The prospective cohort study in three-year olds. Early Hum. Dev..

[22] Jedrychowski W., Perera F.P., Jankowski J., Mrozek-Budzyn D., Mroz E., Flak E., Edwards S., Skarupa A., Lisowska-Miszczyk I. (2009). Very low prenatal exposure to lead and mental development of children in infancy and early childhood: Krakow prospective cohort study. Neuroepidemiology.

[23] Ris M.D., Dietrich K.N., Succop P.A., Berger O.G., Bornschein R.L. (2004). Early exposure to lead and neuropsychological outcome in adolescence. J. Int. Neuropsychol. Soc..

[24] Taylor C.M., Kordas K., Golding J., Emond A.M. (2017). Effects of low-level prenatal lead exposure on child IQ at 4 and 8 years in a UK birth cohort study. Neurotoxicology.

[25] Hu H., Téllez-Rojo M.M., Bellinger D., Smith D., Ettinger A.S., Lamadrid-Figueroa H., Schwartz J., Schnaas L., Mercado-García A., Hernández-Avila M. (2006). Fetal lead exposure at each stage of pregnancy as a predictor of infant mental development. Environ. Health Perspect..

[26] Schnaas L., Rothenberg S.J., Flores M., Martinez S., Hernandez C., Osorio E., Velasco S.R., Perroni E. (2006). Reduced intellectual development in children with prenatal lead exposure. Environ. Health Perspect..

[27] Dietrich K.N., Krafft K.M., Bornschein R.L., Hammond P.B., Berger O., Succop P.A., Bier M. (1987). Low-level fetal lead exposure effect on neurobehavioral development in early infancy. Pediatrics.

[28] Polanska K., Hanke W., Gromadzinska J., Ligocka D., Gulczynska E., Sobala W., Wasowicz W. (2009). Polish mother and child cohort study—Defining the problem, the aim of the study and methodological assumption. Int. J. Occup. Med. Environ. Health.

[29] Polanska K., Hanke W., Jurewicz J., Sobala W., Madsen C., Nafstad P., Magnus P. (2011). Polish mother and child cohort study (REPRO_PL)—Methodology of follow-up of the children. Int. J. Occup. Med. Environ. Health.

[30] Polanska K., Muszynski P., Sobala W., Dziewirska E., Merecz-Kot D., Hanke W. (2015). Maternal lifestyle during pregnancy and child psychomotor development—Polish Mother and Child Cohort Study. Early Hum. Dev..

[31] Polanska K., Krol A., Merecz-Kot D., Ligocka D., Mikołajewska K., Mirabella F., Chiarotti F., Calamandrei G., Hanke W. (2017). Environmental Tobacco Smoke Exposure during Pregnancy and Child Neurodevelopment. Int. J. Environ. Res. Public Health.

[32] Polanska K., Hanke W., Krol A., Gromadzinska J., Kuras R., Janasik B., Wasowicz W., Mirabella F., Chiarotti F., Calamandrei G. (2017). Micronutrients during pregnancy and child psychomotor development: Opposite effects of Zinc and Selenium. Environ. Res..

[33] Goyer R.A. (1990). Transplacental transport of lead. Environ. Health Perspect..

[34] Toews A.D., Kolber A., Hayward J., Krigman M.R., Morell P. (1977). Experimental lead encephalopathy in the suckling rat: Concentration of lead in cellular fractions enriched in brain capillaries. Brain Res..

[35] Bellinger D.C., Matthews-Bellinger J.A., Kordas K. (2016). A developmental perspective on early-life exposure to neurotoxicants. Environ. Int..

[36] Anderson D.W., Mettil W., Schneider J.S. (2016). Effects of low level lead exposure on associative learning and memory in the rat: Influences of sex and developmental timing of exposure. Toxicol. Lett..

[37] Schneider J.S., Anderson D.W., Talsania K., Mettil W., Vadigepalli R. (2012). Effects of developmental lead exposure on the hippocampal transcriptome: Influences of sex, developmental period, and lead exposure level. Toxicol. Sci..

[38] Bunn T.L., Parsons P.J., Kao E., Dietert R.R. (2001). Gender-based profiles of developmental immunotoxicity to lead in the rat: Assessment in juveniles and adults. J. Toxicol. Environ. Health A.

[39] Bunn T.L., Parsons P.J., Kao E., Dietert R.R. (2001). Exposure to lead during critical windows of embryonic development: Differential immunotoxic outcome based on stage of exposure and gender. Toxicol. Sci..

[40] Kasten-Jolly J., Lawrence D.A. (2017). Sex-specific effects of developmental lead exposure on the immune-neuroendocrine network. Toxicol. Appl. Pharmacol..

[41] Miller A.A., De Silva T.M., Jackman K.A., Sobey C.G. (2007). Effect of gender and sex hormones on vascular oxidative stress. Clin. Exp. Pharmacol. Physiol..

[42] Malagutti K.S., da Silva A.P., Campos Braga H., Arruda Mitozo P., Soares dos Santos A.R., Dafre A.L., de Bem A.F., Farina M. (2009). 17β-estradiol decreases methylmercury-induced neurotoxicity in Male mice. Environ. Toxicol. Pharmacol..

[43] McCarthy M.M., Auger A.P., Bale T.L., De Vries G.J., Dunn G.A., Forger N.G., Murray E.K., Nugent B.M., Schwarz J.M., Wilson M.E. (2009). The epigenetics of sex differences in the brain. J. Neurosci..

[44] Numata S., Ye T., Hyde T.M., Guitart-Navarro X., Tao R., Wininger M., Colantuoni C., Weinberger D.R., Kleinman J.E., Lipska B.K. (2012). DNA methylation signatures in development and aging of the human prefrontal cortex. Am. J. Hum. Genet..

[45] Chatterjee A., Lagisz M., Rodger E.J., Zhen L., Stockwell P.A., Duncan E.J., Horsfield J.A., Jeyakani J., Mathavan S., Ozaki Y. (2016). Sex differences in DNA methylation and expression in zebrafish brain: A test of an extended ‘male sex drive’ hypothesis. Gene.

[46] Schwarz J.M., Nugent B.M., McCarthy M.M. (2010). Developmental and hormone-induced epigenetic changes to estrogen and progesterone receptor genes in brain are dynamic across the life span. Endocrinology.

[47] Singh G., Singh V., Wang Z.X., Voisin G., Lefebvre F., Navenot J.M., Evans B., Verma M., Anderson D.W., Schneider J.S. (2018). Effects of developmental lead exposure on the hippocampal methylome: Influences of sex and timing and level of exposure. Toxicol. Lett..

[48] Sen A., Heredia N., Senut M.C., Hess M., Land S., Qu W., Hollacher K., Dereski M.O., Ruden D.M. (2015). Early life lead exposure causes gender-specific changes in the DNA methylation profile of DNA extracted from dried blood spots. Epigenomics.

[49] Engstrom K., Rydbeck F., Kippler M., Wojdacz T.K., Arifeen S., Vahter M., Broberg K. (2015). Prenatal lead exposure is associated with decreased cord blood DNA methylation of the glycoprotein VI gene involved in platelet activation and thrombus formation. Environ. Epigenet..

[50] Singh G., Singh V., Sobolewski M., Cory-Slechta D.A., Schneider J.S. (2018). Sex-Dependent Effects of Developmental Lead Exposure on the Brain. Front. Genet..

[51] Vahter M., Gochfeld M., Casati B., Thiruchelvam M., Falk-Filippson A., Kavlock R., Marafante E., Cory-Slechta D. (2007). Implications of gender differences for human health risk assessment and toxicology. Environ. Res..

