# Supplementary material for: Sex-Dependent Impact of Low-Level Lead Exposure during Prenatal Period on Child Psychomotor Functions
Source: Int J Environ Res Public Health. 2018 Oct 16;15(10):2263. doi: 10.3390/ijerph15102263 (PMC6210236; doi:10.3390/ijerph15102263)
Supplement: Supplementary file 1 [file ijerph-15-02263-s001.pdf]

**Supplementary material:**

**Table S1**. The child and parental characteristics.

| Variables | *N* or mean | % or SD |
|---|---|---|
| Gender of the child (*N*=402) | | |
| Boys | 195 | 48.5 |
| Girls | 207 | 51.5 |
| Birth weight (g); mean, SD (*N*=381) | 3356 | 475 |
| Gestational age (weeks); mean; SD (*N*=402) | 39.2 | 1.4 |
| Breastfeeding (*N*=402) | | |
| Yes | 360 | 89.6 |
| No | 42 | 10.4 |
| Nursery attendance at 12 months (*N*=368) | | |
| Yes | 24 | 6.5 |
| No | 344 | 93.5 |
| Nursery attendance at 24 months (*N*=225) | | |
| Yes | 51 | 22.7 |
| No | 174 | 77.3 |
| Age at Bayley test administration (months) | | |
| At 1 year; mean, SD (*N*=367) | 13.2 | 1.4 |
| At 2 years; mean, SD (*N*=232) | 25.1 | 2.8 |
| Maternal age at delivery (years); mean, SD (*N*=402) | 29.1 | 4.3 |
| Paternal age at child birth (years); mean, SD (*N*=376) | 31.1 | 5.5 |
| Maternal education (*N*=401) | | |
| Primary/vocational | 8 | 2.0 |
| Secondary | 112 | 27.9 |
| University | 281 | 70.1 |
| Paternal education (*N*=393) | | |
| Primary/vocational | 13 | 3.3 |
| Secondary | 206 | 52.4 |
| University | 174 | 44.3 |
| Marital status (*N*=398) | | |
| Married | 307 | 77.1 |
| Unmarried | 91 | 22.9 |
| Maternal employment (*N*=385) | | |
| Employed | 348 | 90.4 |
| Unemployed + Never employed | 37 | 9.6 |
| Socio-economic status (*N*=395) | | |
| High | 88 | 22.3 |
| Medium | 272 | 68.9 |
| Low | 35 | 8.9 |
| Maternal prepregnancy BMI ($kg/m^2$); mean, SD (*N*=394) | 22.6 | 3.7 |
| Type of delivery (*N*=353) | | |
| Cesarean | 129 | 36.5 |
| Vaginal | 224 | 63.5 |
| Alcohol consumption during pregnancy (*N*=340) | | |
| Yes | 30 | 8.8 |
| No | 310 | 91.2 |
| Maternal cotinine level during pregnancy; mean, SD | | |
| $1^{st}$ trimester (*N*=337) | 12.9 | 45.5 |

| | | |
|---|---|---|
| 2nd trimester ($N$=236) | 13.9 | 42.9 |
| 3rd trimester ($N$=300) | 8.9 | 29.6 |
| Maternal smoking during pregnancy (based on cotinine level in saliva in the 1st trimester) ($N$=337) | | |
| Yes (>=10 ng/mL) | 39 | 11.6 |
| No (<10 ng/mL) | 298 | 88.4 |
| ETS child exposure at 1 year of age ($N$=368) | | |
| Yes | 146 | 39.7 |
| No | 222 | 60.3 |
| ETS child exposure at 2 years of age ($N$=232) | | |
| Yes | 86 | 37.1 |
| No | 146 | 62.9 |

**Table S2**. Impact of selected socio-demographic, lifestyle variables and 2nd trimester blood lead level on the child psychomotor development within the first two years of life (*N* subjects = 200, *N* observations = 300).

| Variables | Cognitive β | Cognitive 95%CI | Cognitive *p* | Language β | Language 95%CI | Language *p* | Motor β | Motor 95%CI | Motor *p* |
|---|---|---|---|---|---|---|---|---|---|
| Examiner 1 | −8.36 | −12.04 ; −4.69 | <0.01 | −11.19 | −15.35 ; −7.03 | <0.01 | −14.81 | −18.45 ; −11.17 | <0.01 |
| Examiner 2 | −6.72 | −9.55 ; −3.89 | <0.01 | 0.05 | −3.08 ; 3.17 | 0.98 | 4.33 | 0.82 ; 7.84 | 0.02 |
| Examiner 4 | 17.22 | 11.47 ; 22.98 | <0.01 | −9.26 | −14.13 ; −4.39 | <0.01 | −5.14 | −11.46 ; 1.17 | 0.11 |
| Child age at examination (months) | 1.23 | −0.21 ; 2.67 | 0.09 | −4.88 | −6.28 ; −3.49 | <0.01 | 2.14 | 0.67 ; 3.61 | 0.01 |
| Child gender (male) | 3.07 | 0.11 ; 6.03 | 0.04 | 6.30 | 3.36 ; 9.23 | <0.01 | −0.29 | −3.44 ; 2.86 | 0.86 |
| Blood lead level (μg/dL) | 0.60 | −1.33 ; 2.52 | 0.54 | −0.47 | −2.62 ; 1.67 | 0.66 | −0.28 | −2.94 ; 2.37 | 0.83 |
| Interaction blood lead level (μg/dL) by child age at examination (months) | −1.46 | −2.93 ; 0.01 | 0.05 | −0.72 | −2.35 ; 0.91 | 0.38 | 0.77 | −0.85 ; 2.39 | 0.35 |
| Interaction blood lead level (μg/dL) by child gender | −0.23 | −3.10 ; 2.64 | 0.87 | −0.63 | −3.48 ; 2.22 | 0.66 | −0.58 | −3.93 ; 2.76 | 0.73 |
| Maternal age at delivery (years) | 0.11 | −0.26 ; 0.48 | 0.55 | 0.19 | −0.17 ; 0.54 | 0.30 | −0.09 | −0.46 ; 0.29 | 0.65 |
| Maternal education (high school) | 2.39 | −1.88 ; 6.67 | 0.27 | −2.93 | −13.21 ; 7.34 | 0.57 | −3.70 | −11.11 ; 3.72 | 0.33 |
| Maternal education (university degree or more) | −1.74 | −4.85 ; 1.37 | 0.27 | −3.24 | −6.33 ; −0.16 | 0.04 | −0.51 | −3.76 ; 2.74 | 0.76 |
| Cotinine level during pregnancy (ng/mL) | −0.58 | −1.87 ; 0.70 | 0.37 | 0.39 | −1.02 ; 1.79 | 0.59 | −0.92 | −2.42 ; 0.59 | 0.23 |

2nd trimester lead levels and cotinine level during pregnancy are log10 transformed and standardized; child age at examination is standardized.
Data are reported as β – beta coefficients, 95% CI – 95% confidence interval, *p*-values.

**Table S3**. Impact of selected socio-demographic, lifestyle variables and cord blood lead level on the child psychomotor development within the first two years of life (all subjects; *N* subjects = 238, *N* observations = 365).

| | Cognitive | | | Language | | | Motor | | |
|---|---|---|---|---|---|---|---|---|---|
| Variables | β | 95 % CI | *p* | β | 95 % CI | *p* | β | 95 % CI | *p* |
| Examiner 1 | −9.09 | −12.39 ; −5.79 | <0.01 | −12.94 | −16.47 ; −9.42 | <0.01 | −15.18 | −18.64 ; −11.73 | <0.01 |
| Examiner 2 | −8.14 | −11.00 ; −5.27 | <0.01 | −3.38 | −6.46 ; −0.31 | 0.03 | 6.42 | 3.41 ; 9.43 | 0.00 |
| Examiner 4 | 9.23 | 5.05 ; 13.40 | <0.01 | −16.11 | −20.13 ; −12.08 | <0.01 | −10.72 | −15.56 ; −5.89 | <0.01 |
| Child age at examination (months) | 2.61 | 1.17 ; 4.05 | <0.01 | −2.99 | −4.44 ; −1.54 | <0.01 | 2.63 | 1.21 ; 4.04 | <0.01 |
| Child gender (male) | 4.27 | 1.72 ; 6.82 | <0.01 | 6.85 | 4.12 ; 9.59 | <0.01 | 1.93 | -0.90 ; 4.76 | 0.18 |
| Cord blood lead level (µg/dL) | −1.83 | −3.82 ; 0.15 | 0.07 | −0.39 | −2.63 ; 1.84 | 0.73 | −0.80 | -2.90 ; 1.30 | 0.46 |
| Interaction cord blood lead level (µg/dL) by child age at examination (months) | −1.35 | −2.73 ; 0.03 | 0.06 | −0.20 | −1.71 ; 1.31 | 0.79 | −0.25 | −1.53 ; 1.03 | 0.70 |
| Interaction cord blood lead level (µg/dL) by child gender | 2.09 | -0.41 ; 4.59 | 0.10 | 0.17 | -2.66 ; 3.01 | 0.91 | 1.41 | −1.42 ; 4.23 | 0.33 |
| Maternal age at delivery (years) | 0.07 | −0.26 ; 0.39 | 0.69 | 0.02 | −0.36 ; 0.40 | 0.91 | −0.24 | −0.63 ; 0.14 | 0.21 |
| Maternal education (high school) | 0.93 | −2.23 ; 4.10 | 0.56 | −1.91 | −10.22 ; 6.39 | 0.65 | −8.68 | −13.14 ; −4.22 | <0.01 |
| Maternal education (university degree or more) | −3.05 | −5.96 ; -0.13 | 0.04 | −4.06 | −7.54 ; -0.57 | 0.02 | −0.88 | −4.36 ; 2.60 | 0.62 |
| Cotinine level during pregnancy (ng/mL) | 0.46 | -0.72 ; 1.64 | 0.44 | 0.08 | −1.27 ; 1.42 | 0.91 | −0.35 | −1.55 ; 0.86 | 0.57 |

Cord blood lead levels and cotinine level during pregnancy are log10 transformed and standardized; child age at examination is standardized.
Data are reported as β – beta coefficients, 95% CI – 95% confidence interval, *p*-values.

**Table S4**. Impact of selected socio-demographic, lifestyle variables and cord blood lead level on the child psychomotor development within the first two years of life (excluding subjects with cord blood lead level ≥4.0 μg/dL; *N* subjects = 235, *N* observations = 359).

| Variables | Cognitive | | | Language | | | Motor | | |
|---|---|---|---|---|---|---|---|---|---|
| | β | 95%CI | *p* | β | 95%CI | *p* | β | 95%CI | *p* |
| Examiner 1 | −9.50 | −12.79 ; −6.21 | <0.01 | −13.37 | −16.84 ; −9.90 | <0.01 | −15.84 | −19.13 ; −12.55 | <0.01 |
| Examiner 2 | −8.10 | −11.02 ; −5.19 | <0.01 | −3.14 | −6.23 ; −0.06 | 0.05 | 6.27 | 3.23 ; 9.31 | <0.01 |
| Examiner 4 | 9.11 | 4.96 ; 13.26 | <0.01 | −16.22 | −20.26 ; −12.19 | <0.01 | −10.93 | −15.74 ; −6.12 | <0.01 |
| Child age at examination (months) | 2.54 | 1.10 ; 3.98 | <0.01 | -3.09 | −4.55 ; −1.64 | <0.01 | 2.55 | 1.13 ; 3.97 | <0.01 |
| Child gender (male) | 4.31 | 1.77 ; 6.86 | <0.01 | 6.89 | 4.12 ; 9.66 | <0.01 | 1.83 | −1.01 ; 4.66 | 0.21 |
| Cord blood lead level (μg/dL) | −2.41 | −4.38 ; −0.43 | 0.02 | −0.68 | −3.13 ; 1.76 | 0.58 | −1.17 | −3.44 ; 1.09 | 0.31 |
| Interaction cord blood lead level (μg/dL) by child age at examination (months) | −1.60 | −3.45 ; 0.26 | 0.09 | −0.59 | −2.36 ; 1.18 | 0.51 | −0.42 | −1.76 ; 0.92 | 0.54 |
| Interaction cord blood lead level (μg/dL) by child gender | 2.22 | −0.48 ; 4.91 | 0.11 | 0.15 | −2.96 ; 3.26 | 0.92 | 0.98 | −2.23 ; 4.19 | 0.55 |
| Maternal age at delivery (years) | 0.06 | −0.27 ; 0.39 | 0.72 | −0.01 | −0.39 ; 0.37 | 0.97 | −0.25 | −0.64 ; 0.15 | 0.22 |
| Maternal education (high school) | 0.76 | −2.42 ; 3.93 | 0.64 | −2.24 | −10.71 ; 6.23 | 0.60 | −8.84 | −13.55 ; -4.13 | <0.01 |
| Maternal education (university degree or more) | −3.46 | −6.39 ; −0.54 | 0.02 | −4.61 | −8.16 ; −1.05 | 0.01 | −1.12 | −4.70 ; 2.47 | 0.54 |
| Cotinine level during pregnancy (ng/mL) | 0.61 | −0.57 ; 1.79 | 0.31 | 0.24 | −1.13 ; 1.61 | 0.73 | −0.20 | −1.43 ; 1.02 | 0.74 |

Cord blood lead levels and cotinine level during pregnancy are log10 transformed and standardized; child age at examination is standardized.
Data are reported as β – beta coefficients, 95% CI – 95% confidence interval, *p*-values

**Table S5.** Impact of selected socio-demographic, lifestyle variables and cord blood lead level on the child psychomotor development within the first two years of life (excluding subjects with cord blood lead level ≥3.0 μg/dL; *N* subjects = 233, *N* observations = 356).

| Variables | Cognitive | | | Language | | | Motor | | |
|---|---|---|---|---|---|---|---|---|---|
| | β | 95%CI | *p* | β | 95%CI | *p* | β | 95%CI | *p* |
| Examiner 1 | −9.55 | −12.81 ; −6.28 | <0.01 | −13.36 | −16.83 ; −9.89 | <0.01 | −15.83 | −19.13 ; −12.53 | <0.01 |
| Examiner 2 | −8.01 | −10.95 ; −5.08 | <0.01 | −3.05 | −6.14 ; 0.05 | 0.05 | 6.30 | 3.24 ; 9.37 | <0.01 |
| Examiner 4 | 9.12 | 4.93 ; 13.31 | <0.01 | −15.72 | −19.68 ; −11.75 | <0.01 | −10.89 | −15.76 ; −6.02 | <0.01 |
| Child age at examination (months) | 2.38 | 0.96 ; 3.81 | <0.01 | −3.24 | −4.69 ; −1.80 | <0.01 | 2.53 | 1.10 ; 3.96 | <0.01 |
| Child gender (male) | 4.28 | 1.74 ; 6.82 | <0.01 | 6.98 | 4.21 ; 9.74 | <0.01 | 1.82 | −1.03 ; 4.67 | 0.21 |
| Cord blood lead level (μg/dL) | −2.48 | −4.46 ; −0.51 | 0.01 | −0.74 | −3.20 ; 1.73 | 0.56 | −1.18 | −3.45 ; 1.08 | 0.30 |
| Interaction cord blood lead level (μg/dL) by child age at examination (months) | −2.37 | −3.94 ; −0.80 | <0.01 | −1.31 | −3.03 ; 0.41 | 0.13 | -0.54 | −2.05 ; 0.98 | 0.48 |
| Interaction cord blood lead level (μg/dL) by child gender | 2.08 | −0.76 ; 4.93 | 0.15 | 0.40 | −2.71 ; 3.51 | 0.80 | 0.93 | −2.52 ; 4.39 | 0.59 |
| Maternal age at delivery (years) | 0.06 | −0.28 ; 0.39 | 0.74 | 0.01 | −0.38 ; 0.39 | 0.97 | −0.25 | −0.65 ; 0.15 | 0.22 |
| Maternal education (high school) | 0.74 | −2.47 ; 3.95 | 0.65 | −2.16 | −10.67 ; 6.35 | 0.62 | −8.86 | −13.61 ; -4.10 | <0.01 |
| Maternal education (university degree or more) | −3.64 | −6.60 ; −0.68 | 0.02 | −4.96 | −8.55 ; −1.38 | 0.01 | −1.19 | −4.82 ; 2.45 | 0.52 |
| Cotinine level during pregnancy (ng/mL) | 0.65 | −0.53 ; 1.84 | 0.28 | 0.27 | −1.12 ; 1.65 | 0.70 | −0.19 | −1.43 ; 1.05 | 0.76 |

Cord blood lead levels and cotinine level during pregnancy are log10 transformed and standardized; child age at examination is standardized.
Data are reported as β – beta coefficients, 95% CI – 95% confidence interval, *p*-values.

**Figure S1**. Scatterplots relating blood lead levels in the 2nd trimester of pregnancy and in the cord blood to the Bayley cognitive, language and motor scores at one and two years of age.

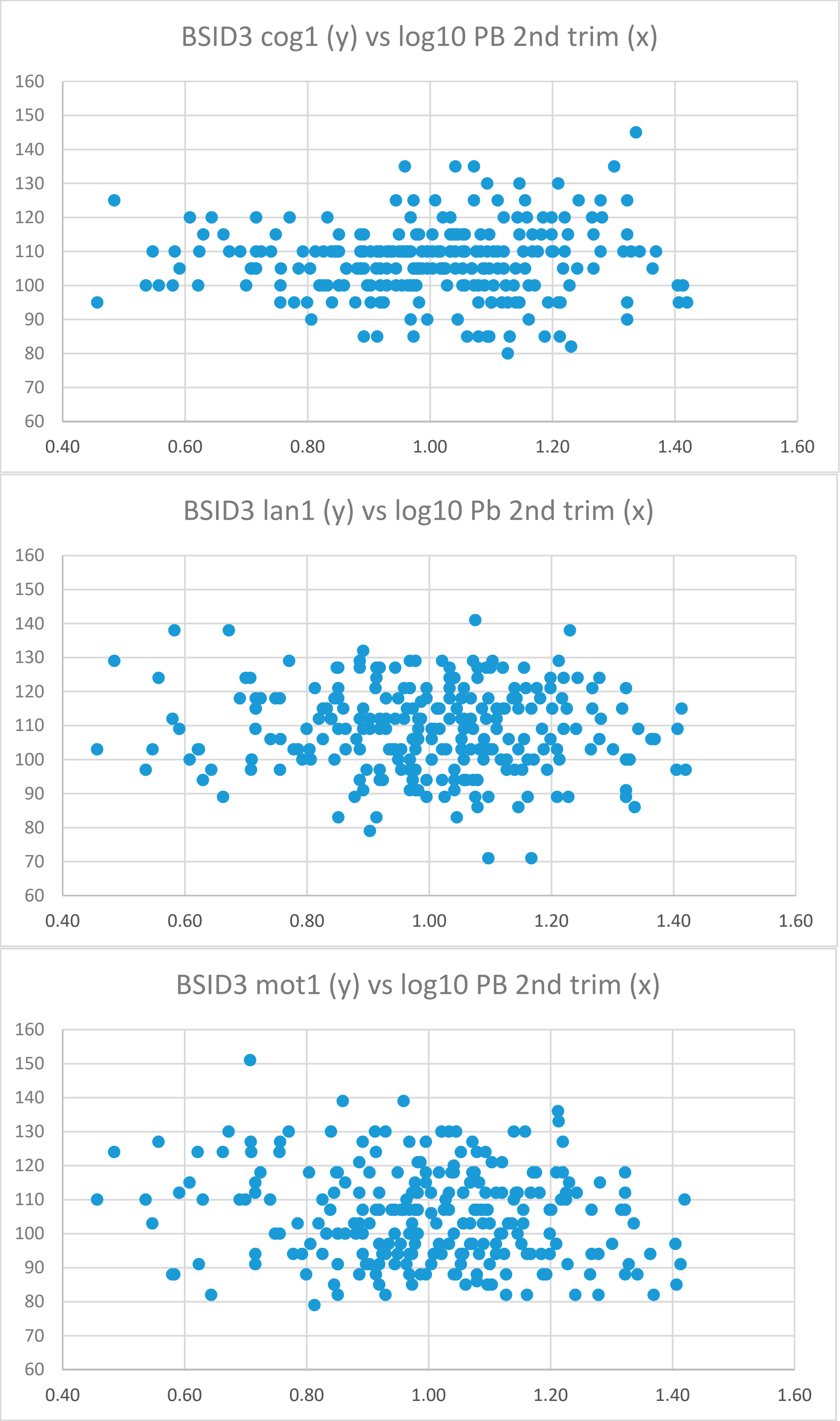

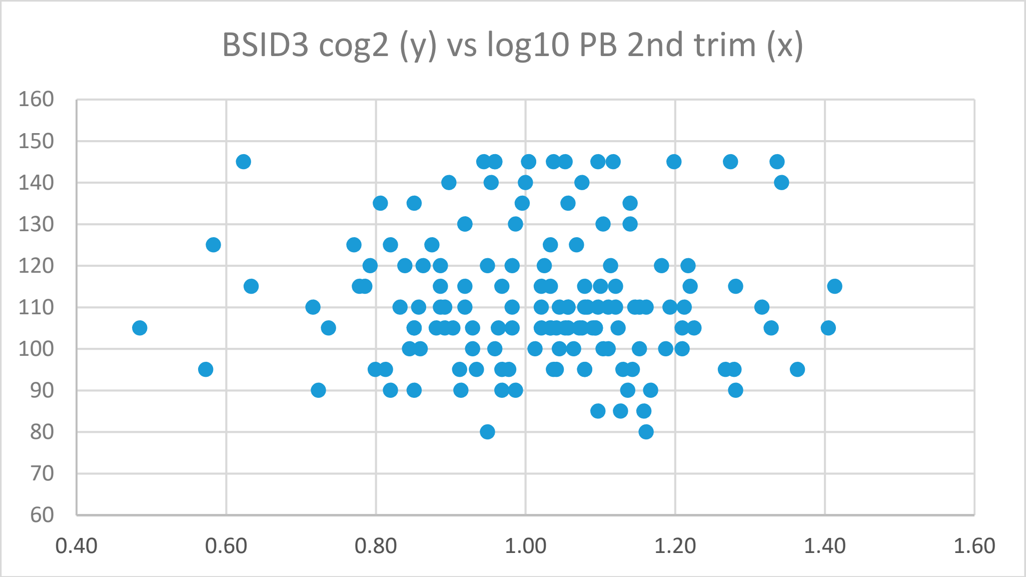

BSID3 cog2 (y) vs log10 PB 2nd trim (x)
160
150
140
130
120
110
100
90
80
70
60
0.40
0.60
0.80
1.00
1.20
1.40
1.60


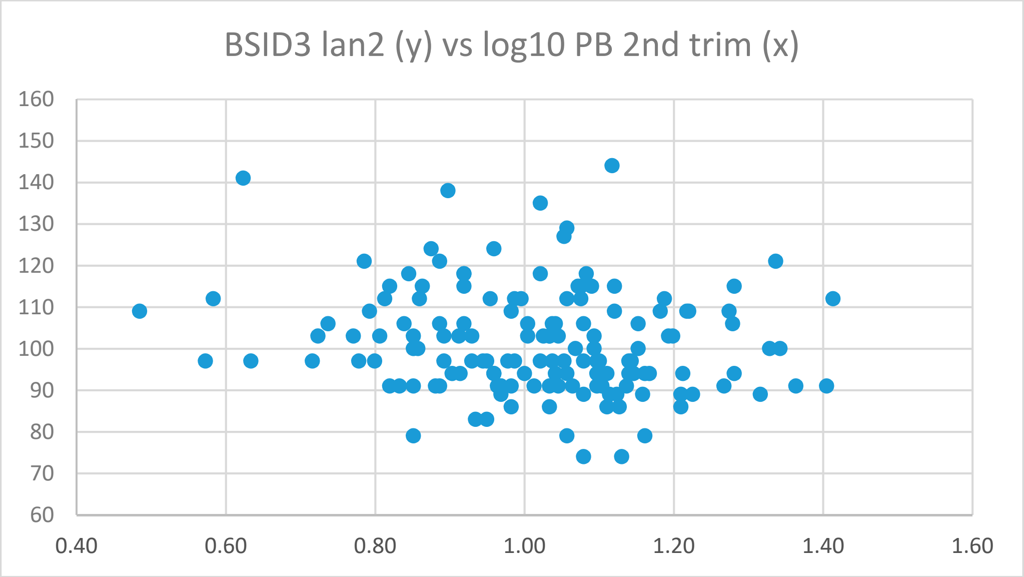

BSID3 lan2 (y) vs log10 PB 2nd trim (x)
160
150
140
130
120
110
100
90
80
70
60
0.40
0.60
0.80
1.00
1.20
1.40
1.60


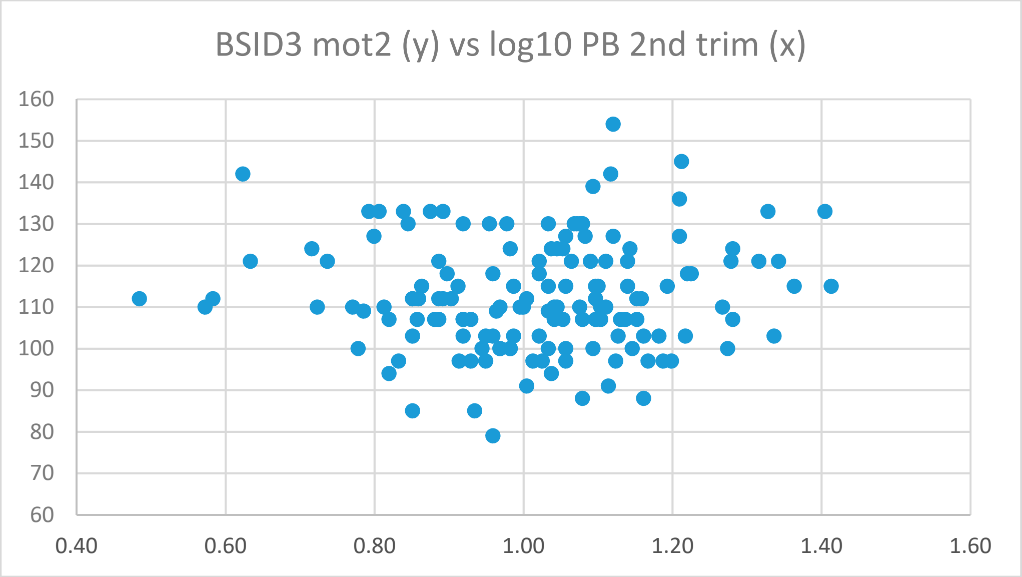

BSID3 mot2 (y) vs log10 PB 2nd trim (x)
160
150
140
130
120
110
100
90
80
70
60
0.40
0.60
0.80
1.00
1.20
1.40
1.60

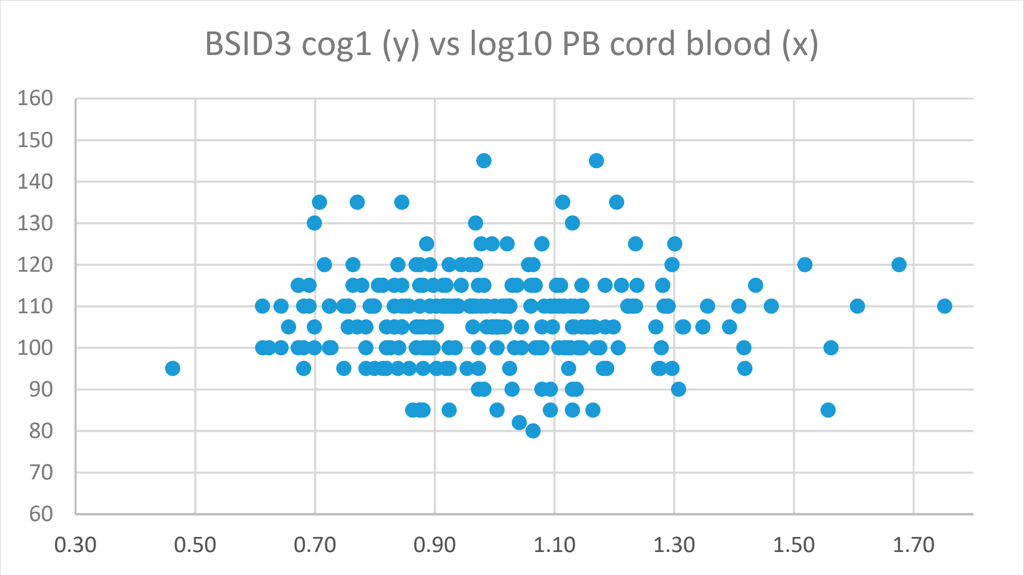
BSID3 cog1 (y) vs log10 PB cord blood (x)
160
150
140
130
120
110
100
90
80
70
60
0.30
0.50
0.70
0.90
1.10
1.30
1.50
1.70

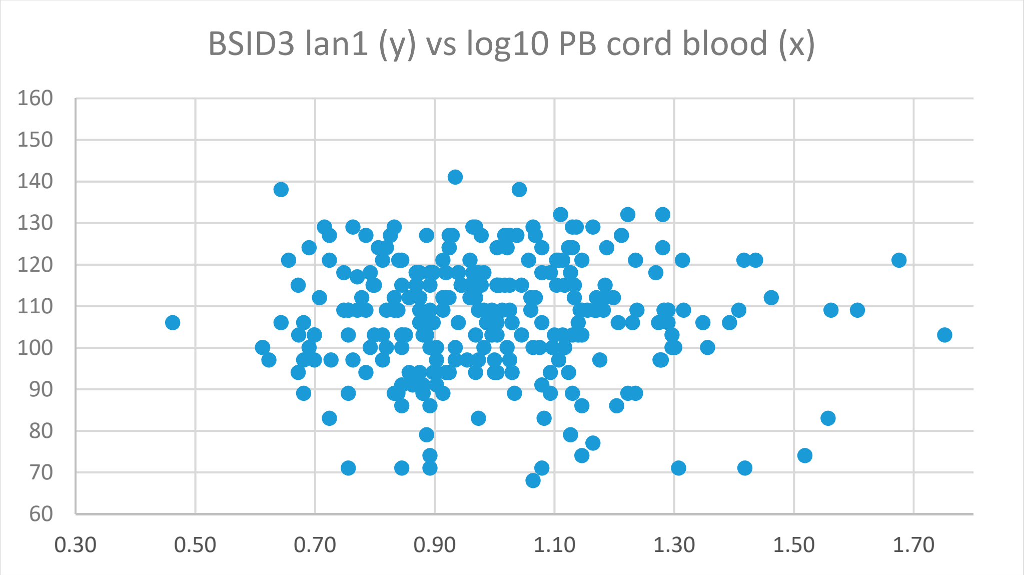
BSID3 lan1 (y) vs log10 PB cord blood (x)
160
150
140
130
120
110
100
90
80
70
60
0.30
0.50
0.70
0.90
1.10
1.30
1.50
1.70

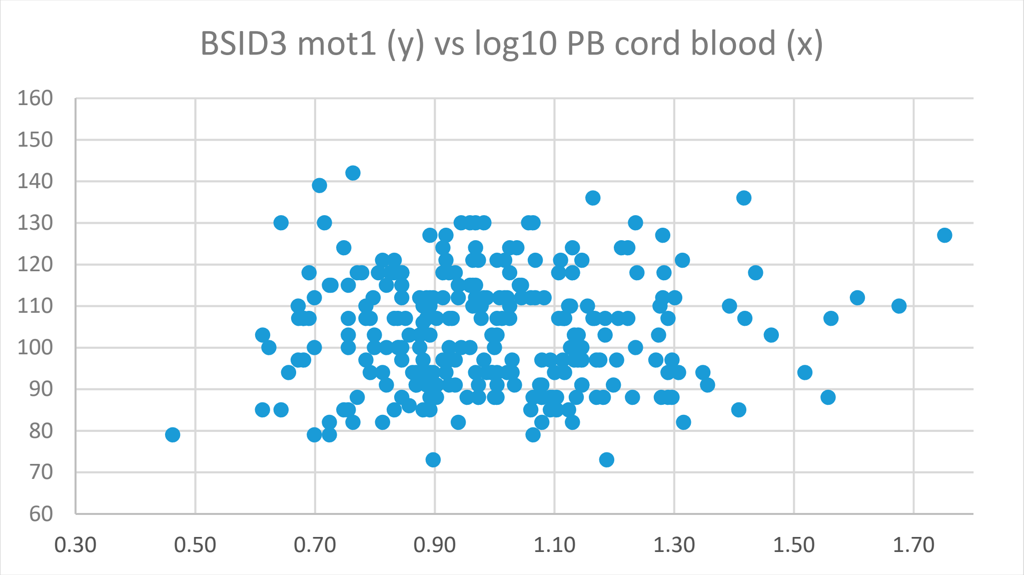
BSID3 mot1 (y) vs log10 PB cord blood (x)
160
150
140
130
120
110
100
90
80
70
60
0.30
0.50
0.70
0.90
1.10
1.30
1.50
1.70

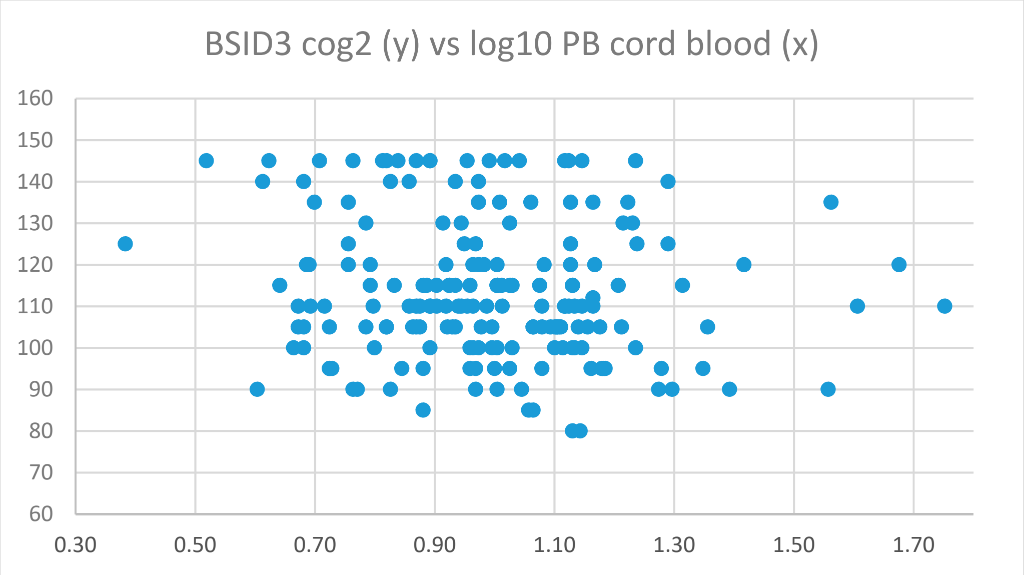
BSID3 cog2 (y) vs log10 PB cord blood (x)
160
150
140
130
120
110
100
90
80
70
60
0.30
0.50
0.70
0.90
1.10
1.30
1.50
1.70

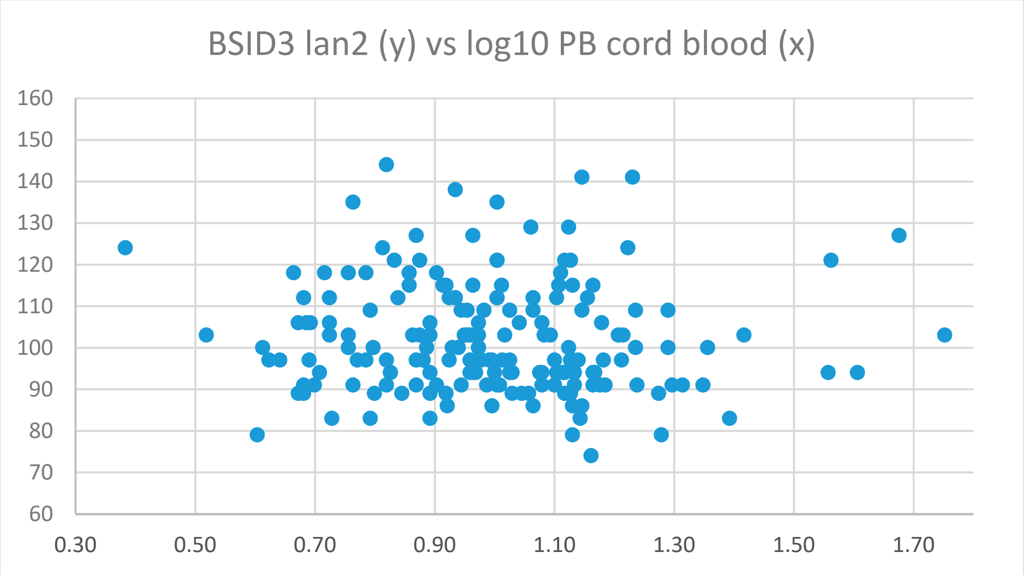
BSID3 lan2 (y) vs log10 PB cord blood (x)
160
150
140
130
120
110
100
90
80
70
60
0.30
0.50
0.70
0.90
1.10
1.30
1.50
1.70

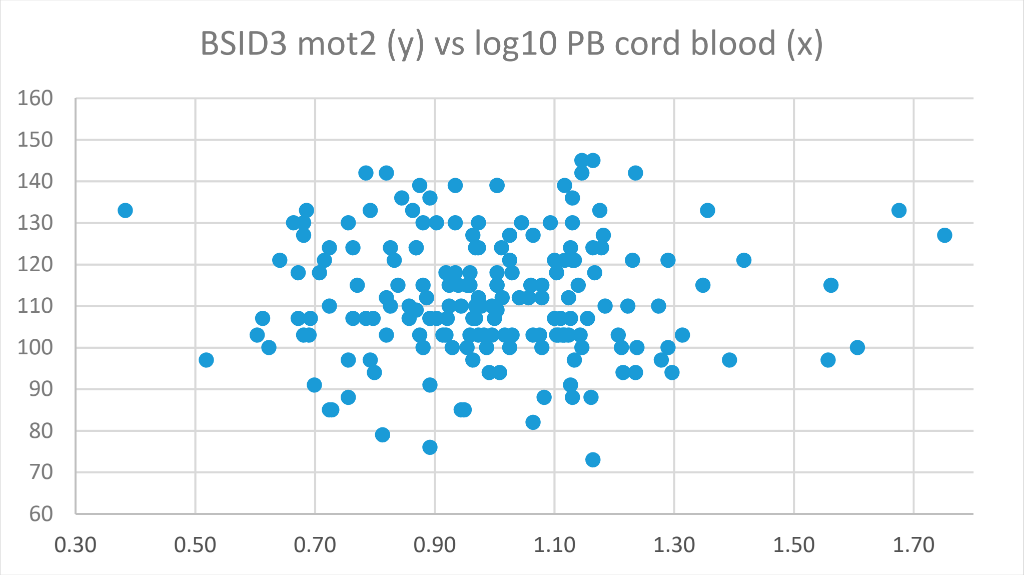
BSID3 mot2 (y) vs log10 PB cord blood (x)
160
150
140
130
120
110
100
90
80
70
60
0.30
0.50
0.70
0.90
1.10
1.30
1.50
1.70

**Figure S2.** Point estimates and 95% confidence intervals of regression coefficients measuring the effect of blood lead level (log10-transformed and standardized) on Bayley scores in the subgroups of children based on quartiles of blood lead level.

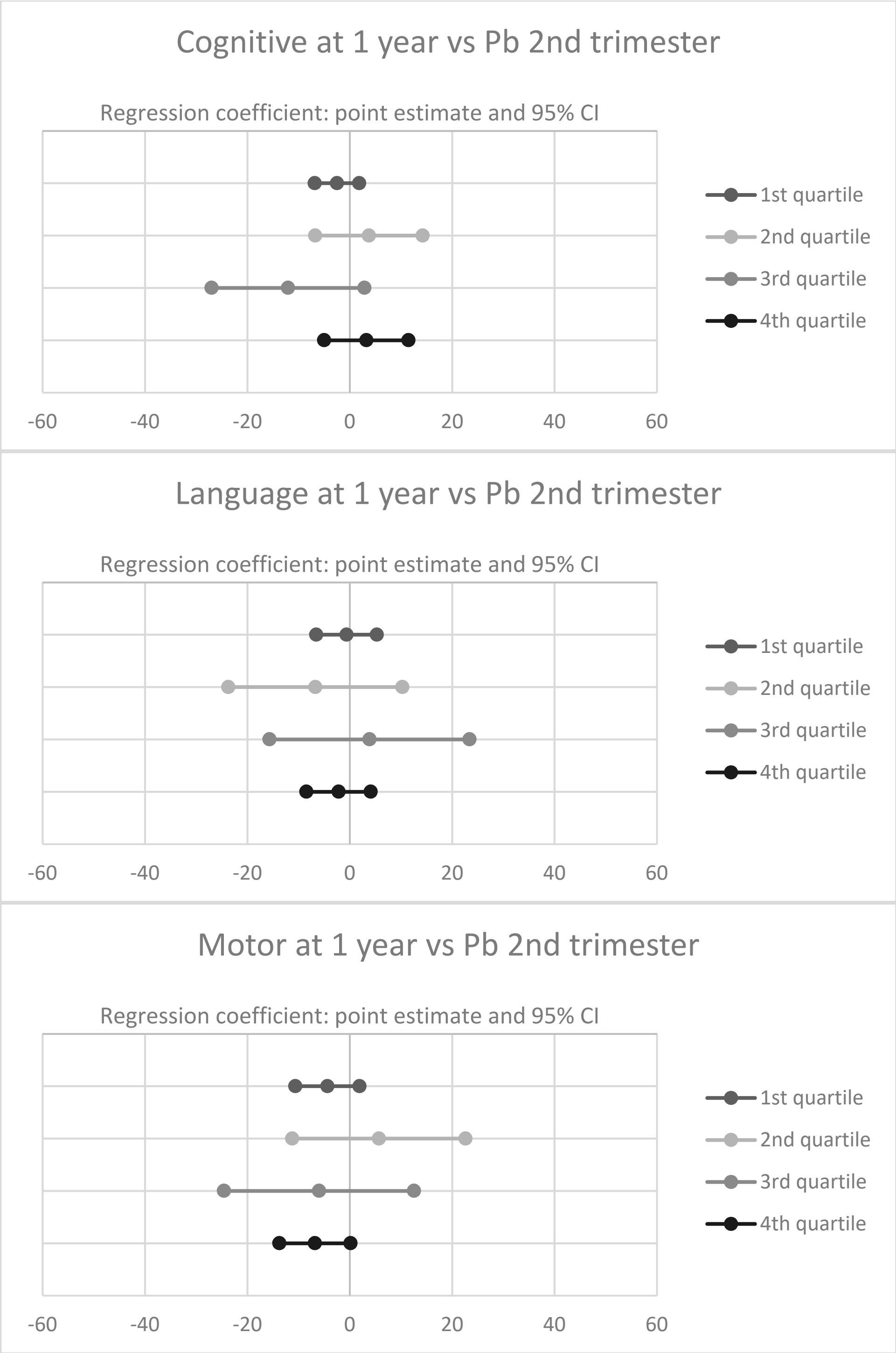

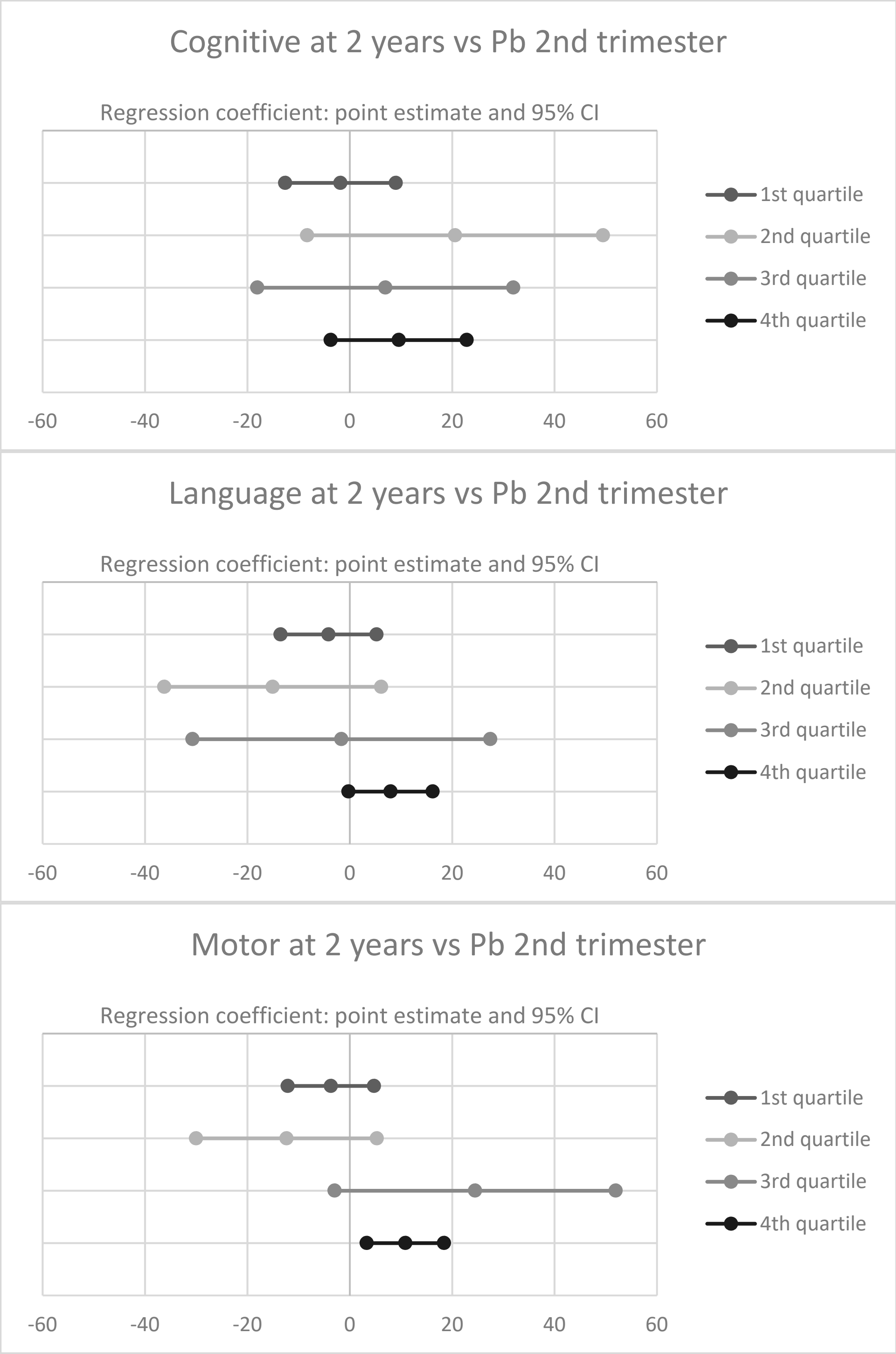
Cognitive at 2 years vs Pb 2nd trimester
Regression coefficient: point estimate and 95% CI
-60
-40
-20
0
20
40
60
1st quartile
2nd quartile
3rd quartile
4th quartile
Language at 2 years vs Pb 2nd trimester
Regression coefficient: point estimate and 95% CI
-60
-40
-20
0
20
40
60
1st quartile
2nd quartile
3rd quartile
4th quartile
Motor at 2 years vs Pb 2nd trimester
Regression coefficient: point estimate and 95% CI
-60
-40
-20
0
20
40
60
1st quartile
2nd quartile
3rd quartile
4th quartile

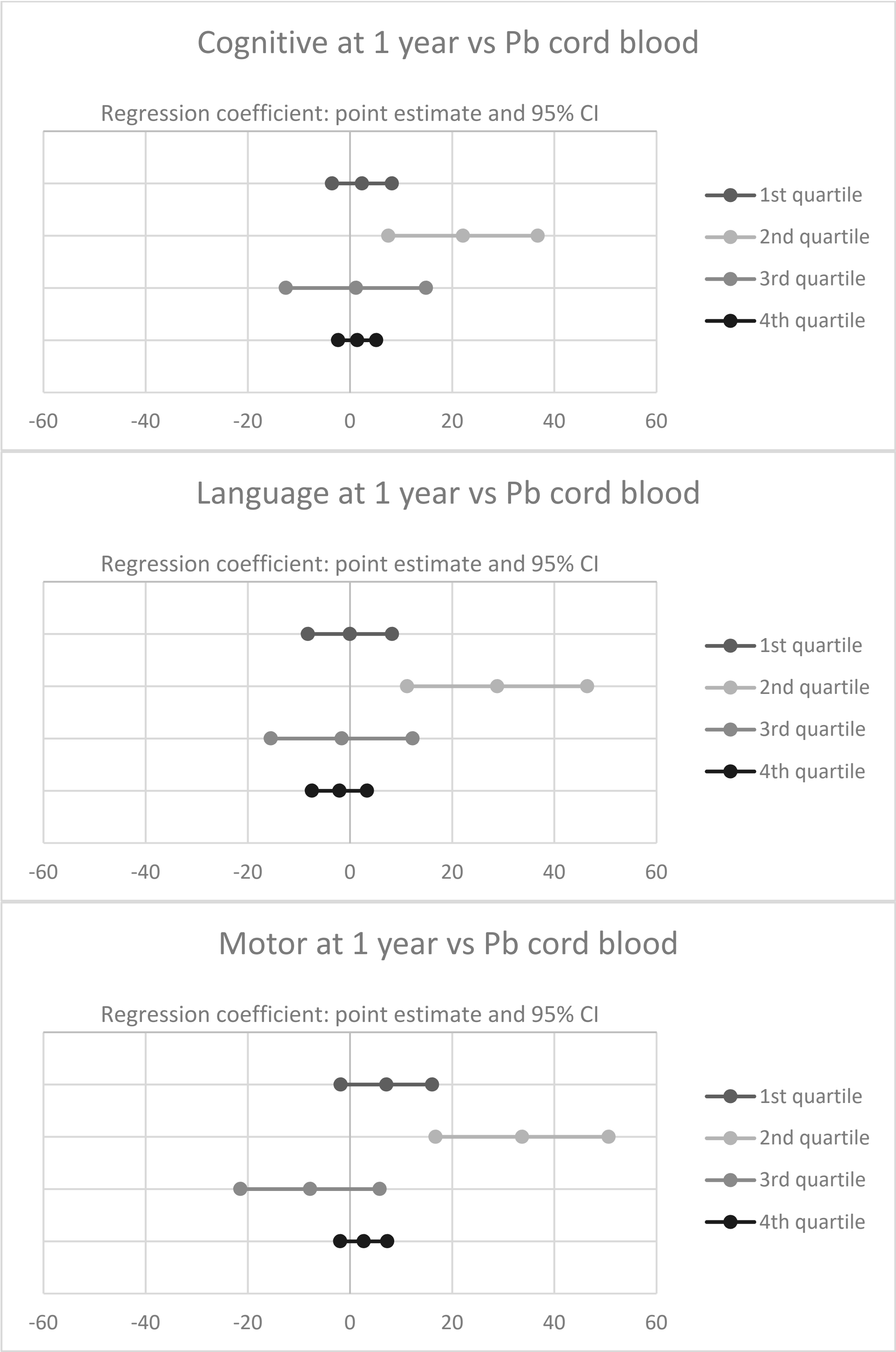

Cognitive at 1 year vs Pb cord blood
Regression coefficient: point estimate and 95% CI
-60 -40 -20 0 20 40 60
1st quartile
2nd quartile
3rd quartile
4th quartile
Language at 1 year vs Pb cord blood
Regression coefficient: point estimate and 95% CI
-60 -40 -20 0 20 40 60
1st quartile
2nd quartile
3rd quartile
4th quartile
Motor at 1 year vs Pb cord blood
Regression coefficient: point estimate and 95% CI
-60 -40 -20 0 20 40 60
1st quartile
2nd quartile
3rd quartile
4th quartile

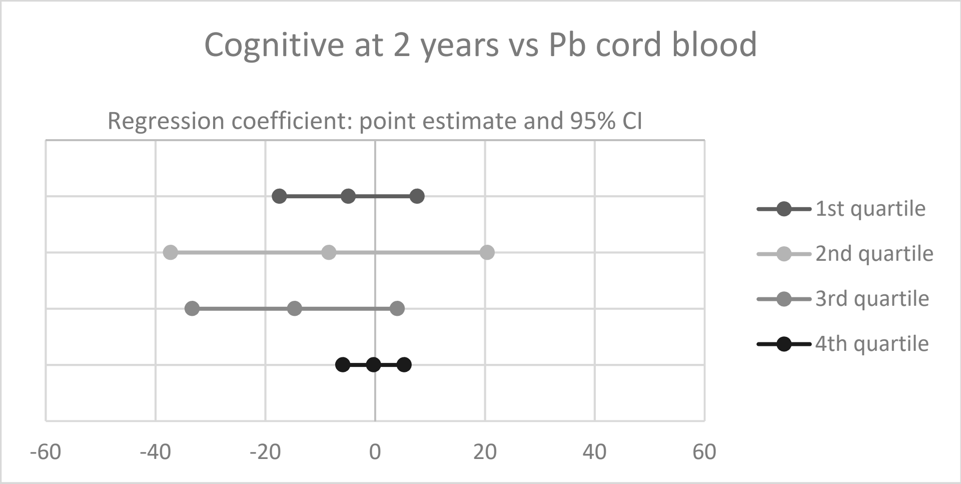

Cognitive at 2 years vs Pb cord blood
Regression coefficient: point estimate and 95% CI
-60
-40
-20
0
20
40
60
1st quartile
2nd quartile
3rd quartile
4th quartile


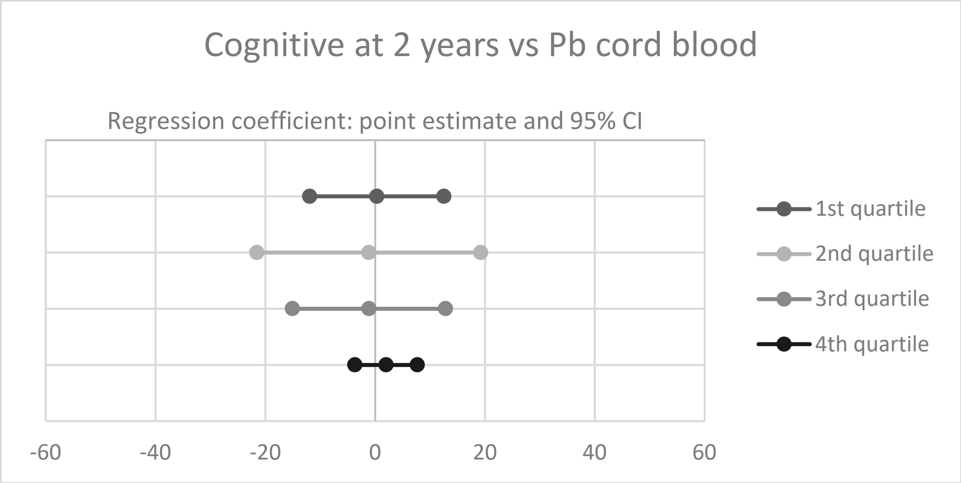

Cognitive at 2 years vs Pb cord blood
Regression coefficient: point estimate and 95% CI
-60
-40
-20
0
20
40
60
1st quartile
2nd quartile
3rd quartile
4th quartile


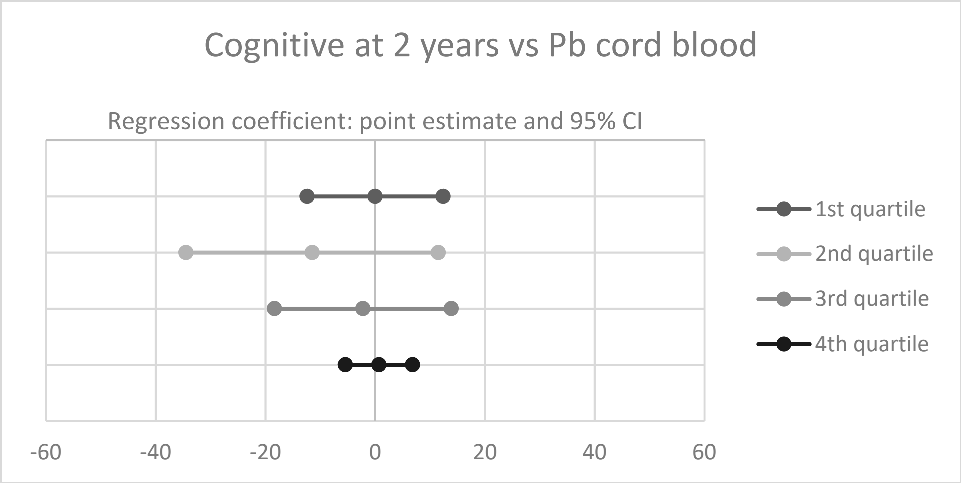

Cognitive at 2 years vs Pb cord blood
Regression coefficient: point estimate and 95% CI
-60
-40
-20
0
20
40
60
1st quartile
2nd quartile
3rd quartile
4th quartile
